# Reconfigurable, non-volatile neuromorphic photovoltaics

**DOI:** 10.1038/s41377-023-01226-y

**Published:** 2023-07-27

**Authors:** Antoni Rogalski

**Affiliations:** Institute of Applied Physics, Military University of Technology, Warsaw, Poland

**Keywords:** Optics and photonics, Physics

Nature Nanotechnology (2023)

10.1038/s41565-023-01446-8

The real-time image processing proliferation places specific information computing and energy conservation demands on sensor-rich platforms. Fabrication of reconfigurable image sensors allowing for in-sensory computing usually requires a sophisticated architecture of multiple layers integration. A team of researchers from the State Key Laboratory of Infrared Physics at Shanghai Institute of Technical Physics, Chinese Academy of Sciences in China and from Department of Electrical and Systems Engineering at University of Pennsylvania in United States, has found a very promising approach that integrating sensing, memory, and computing within a simple two-terminal metal/semiconductor/metal architecture. Such neuromorphic photovoltaic detectors driven by S vacancy migration in 2D MoS_2_ can memorize the reconfigurable photoresponse weight and perform the calculation using the electricity generates in situ. Furthermore, scaling the neuromorphic hardware to larger dimensions is conceptually feasible due to the simple device structure and provides various training possibilities for neuromorphic vision applications. Further work on ionic-optical-electronic coupled device can be applied in other semiconducting materials to develop neuromorphic photonic chips.

